# Non-parametric prediction of brain MRI microstructure using transfer learning

**DOI:** 10.1162/imag_a_00548

**Published:** 2025-04-30

**Authors:** Gustavo Chau Loo Kung, Emmanuelle M.M. Weber, Ankita Batra, Lijun Ni, Michael Zeineh, Akshay Chaudhari, Ehsan Adeli, Juliet K. Knowles, Jennifer A. McNab

**Affiliations:** Department of Bioengineering, Stanford University, Stanford, CA, United States; Department of Radiology, Stanford University, Stanford, CA, United States; Department of Neurology, Stanford University, Stanford, CA, United States; Department of Psychiatry and Behavioral Sciences, Stanford University, Stanford, CA, United States; Department of Computer Science, Stanford University, Stanford, CA, United States

**Keywords:** MRI, microstructure, transfer learning, modeling

## Abstract

Magnetic resonance imaging (MRI) can be sensitive to tissue microstructural features and infer parameterized features by performing a voxel-wise fit of the signal to a biophysical model. However, biophysical models rely on simplified representations of brain tissue. Machine learning (ML) techniques may serve as a data-driven approach to optimize for microstructural feature extraction. Unfortunately, training an ML model for these applications requires a large database of paired specimen MRI and histology datasets, which is costly, cumbersome, and challenging to acquire. In this work, we present a novel approach allowing a reliable estimation of brain tissue microstructure using MRI as inputs, with a minimal amount of paired MRI-histology data. Our method involves pretraining a conditional normalizing flow model to predict the distribution of microstructural features. The model is trained on synthetic MRI data generated from unpaired histology and MRI physics, reducing the data requirement in future steps. The synthetic MRI generation data combines segmentation of a publicly available EM slice, feature extraction and MRI simulators. Subsequently, the model is fine-tuned using experimental MRI/Electron Microscopy (EM) data of nine excised mouse brains through transfer learning. This approach enables the prediction of non-parameterized joint distributions of g-ratio and axon diameters for a given voxel based on MRI input. Results show a close agreement between the distributions predicted by the network and the EM ground-truth histograms (mean Jensen-Shannon Distances of 0.24 and 0.23 on the test set, for axon diameter and g-ratios respectively, compared to distances of 0.18 and 0.18 of a direct fitting of a Gamma distribution to the ground truth). The approach also shows up to 4% decreased mean percent errors of the distributions compared to biophysical model fitting and increased prediction capabilities that are consistent with electron microscopy validation and previous biological studies. For example, g-ratio values predicted along the corpus callosum anterior-posterior axis show a significant difference for mice after myelin remodeling seizures are well established (p<0.001) but not before seizure onset (p = 0.562). The results suggest that pretraining on synthetic MRI and then using transfer learning is an effective approach for addressing the lack of paired MRI/histology data when training ML models for microstructure prediction. This approach is a step toward developing a versatile and widely used foundation model for predicting microstructural features using MRI.

## 1 Introduction

Developing non-invasive methods to reliably map features of brain tissue microstructure is valuable for early diagnosis of neurological diseases and gaining insight into brain mechanisms in both health and disease. Magnetic Resonance Imaging (MRI) provides images at millimeter-scale resolution with excellent gray/white matter contrast but also has a unique ability to sensitize the signal to microstructural components within each voxel. Diffusion-weighted MRI (dMRI) and magnetization transfer (MT) are examples of pulse sequences with specific signal representation that enable a non-invasive sensitivity to changes in tissue microstructure (Afzali et al., 2021;Alexander et al., 2019;Does, 2018;Kabani et al., 2002;Sled, 2018). While dMRI is an indirect measure of tissue microstructure through the measurement of the diffusion of water molecules, MT utilizes the exchange of magnetization between free water protons and macromolecular-bound protons to extract microstructural information (Afzali et al., 2021;Alexander et al., 2019;Does, 2018;Kabani et al., 2002;Sled, 2018).

While MRI serves as an indirect measure of tissue microstructure, advanced modeling enable the inference of underlying biophysical characteristics by performing a voxel-wise fit of the MRI signal. Such models have proven successful in extracting complex brain microstructural features such as axon diameter (Alexander et al., 2019;Assaf et al., 2008;Huang et al., 2020;McNab et al., 2013;Veraart et al., 2021), water compartments (Callaghan et al., 1979;Fieremans et al., 2011;Jelescu et al., 2022;Jespersen et al., 2007;Novikov et al., 2018;Reisert et al., 2017;H. Zhang et al., 2012), myelin content (Khormi et al., 2023;van der Weijden et al., 2021), or g-ratio (Chau Loo Kung et al., 2023;Mohammadi & Callaghan, 2021;Stikov et al., 2015;West, Kelm, Carson, Alexander, et al., 2018), which is the ratio of the inner radius of the axon to the outer radius including the myelin sheath, and is an important feature in assessing myelination.

Many widely-used biophysical models rely on simplified representations of brain tissue microstructures and/or the resultant water diffusion behavior, using multi-compartments and geometric shapes. One of the earliest examples of this is the Diffusion Tensor Imaging (DTI) (Basser et al., 1994) which represents the preferred orientations of diffusion in a voxel using a tensor. More sophisticated models such as NODDI (H. Zhang et al., 2012), AxCaliber (Assaf et al., 2008), and White Matter Tract Integrity (Fieremans et al., 2013) rely on a multi-compartment model. In these representations, each compartment is associated with specific intra- and extracellular spaces modeled with cylinders, sticks, spheres, and/or tensors. Accurate estimation of the underlying structure relies on a modeling trade-off: models that are too simple lead to underfitting and thereby fail to capture tissue complexity, whereas models that are too complex can misrepresent the true tissue microstructure due to overfitting (Jelescu et al., 2020;Rokem et al., 2015). Due to these modeling challenges, there is often a desire to compare the model outputs to ground-truth: histology data on the same specimen, acquired using electron or optical microscopy (Berman et al., 2018;Foxley et al., 2021;Leuze et al., 2021;Rusu et al., 2015;Stikov et al., 2015). However, this comparison process can be both costly and challenging due to the scale gap between the imaging modalities (Foxley et al., 2021;Helmstaedter, 2013). Moreover, tissue deformation and degradation during histological processes pose additional obstacles to performing 3D reconstruction of microscopy data, further complicating their registration with MRI data (Alyami et al., 2022;E. Gibson et al., 2013). These challenges ultimately contribute to the scarcity of paired MRI/histology datasets, which are both precious and difficult to obtain.

Machine learning (ML) techniques can enable a new data-driven approach to improve microstructural feature extraction from MRI data. In particular, ML has the potential to i) accelerate and improve the accuracy of existing biophysical fits (de Almeida Martins et al., 2021;Ye, 2017) and ii) generate alternative, more flexible modeling schemes (Chen et al., 2023;Lin et al., 2019). Training an ML model is normally done in a supervised manner, where a pair of inputs and target outputs are fed to the network and a large number of samples are required for the model to generalize correctly. However, obtaining a large amount of experimental paired data (MRI and histology data obtained from the same specimen) to train models for brain microstructure extraction is both costly and challenging to acquire.

Considering the scarcity of paired MRI/histology data, a new study, focused on developing a method for generating realistic 2D white matter models, leveraged simulated EM and MRI data to train a deep learning model for predicting voxel-wise aggregated values of microstructural features (Hédouin et al., 2021). Other studies have leveraged either publicly available histological images or tracing data to create a ground truth, subsequently used to train a supervised deep learning model to predict either the histology contrast (Liang et al., 2022) or fiber orientation dispersion with a higher accuracy than the current state-of-the-art method (Liang et al., 2023).Nath et al. (2019)trained a neural network to predict the orientation distribution function more accurately using a paired MRI/histology dataset of three squirrel monkey brains. Additionally, several recent works have adopted the use of ML techniques trained on synthetic data to replace conventional fitting approaches. For example,Nedjati-Gilani et al. (2017)trained a random forest regressor on simulations to learn a mapping between features derived from diffusion-weighted MR signals and ground-truth microstructure parameters, such as residence time of water inside axons.Palombo et al. (2020)andIanuş et al. (2022)used random forests to fit different parameters of the Soma and Neurite Density MRI (SANDI) model.Diao and Jelescu (2023)used a recursive neural network to predict parameters of the white matter tract integrity (WMTI)-Watson model from the diffusion and kurtosis tensors. They trained the model on simulations and then adapted it to rat and human MRI data via an embedding layer.Fang et al. (2023)proposed a framework to train supervised multilayer perceptrons on synthetic diffusion MRI data derived from neuronal meshes and the Bloch-Torrey equation, enabling the prediction of microstructural features like soma and neurite volume fractions. This approach has been then tested on the MGH Connectome Diffusion Microstructure Dataset. However, to our knowledge, no study has trained a ML model on paired MRI/histology data that was acquired on the same samples for the purpose of predicting microstructural features directly from the MRI data.

In clinical imaging, transfer learning has emerged as a promising technique to address the issue of data scarcity (Cheplygina et al., 2019;Gifani & Shalbaf, 2024;Tajbakhsh et al., 2020;Wakili et al., 2022). This approach consists in leveraging knowledge gained from pretrained models on large datasets and applying it to new, smaller datasets, significantly reducing the amount of data required. Transfer learning has the potential to improve model performance and generalization, utilizing features learned from related tasks while minimizing amount of in-domain training samples.

Our long-term goal would be to build a foundation model that combines prior biophysical model information with data-driven approaches that can be fine tuned for specific applications. Here, we take the first step in this direction by proposing to leverage transfer learning for reliable modeling of the relationship between MRI data and microstructural features with a new data-limited ML approach. Summary statistics from a real EM image of a canine spinal cord (Vuong et al., 2017) were used to generate corresponding synthetic MRI data. These data are employed to train a conditioned normalizing flow model (Rezende & Mohamed, 2015) to predict non-parametric joint distributions of g-ratio and axon diameters from the MT and diffusion MRI values of a given voxel. We utilize paired MRI/EM data from three regions along the mouse corpus callosum to fine-tune the pretrained network through transfer learning. Prior knowledge from biophysical models is used to guide the training process by concatening values of biophysical maps to the context vector that conditions the Normalizing Flow Model (NFM). This approach allows for i) evaluation of model generalization to experimental data, ii) insights from established modeling techniques to be leveraged, and iii) extraction of additional metrics from higher statistics, providing a more comprehensive understanding of microstructural properties. Furthermore, different key design choices in the architecture are also explored, showing that the inclusion of pre-training, biophysical maps, and multi-task learning are necessary for a final good performance of the model.

## 2 Methods

### 2.1 Generation of synthetic MRI

Electron Microscopy (EM) images were obtained from a publicly available dataset (https://osf.io/yp4qg/), which corresponded to single slices of canine spinal cord (Vuong et al., 2017). MRI scans of the canine tissue sample were also included with this dataset and provided a reference to compare synthetic MRI data against. The method for generating histological-derived synthetic MRI is depicted inFigure 1. The histological image was divided into 125 x 125μmtiles representing the regions corresponding to the MRI voxels. The axon and myelin within each tile were segmented using AxonDeepSeg (Zaimi et al., 2018) and AxonDeepSeg’s automatic labeling was used to obtain lists of g-ratios and axon diameters. AxonDeepSeg approximates the axons as ellipses and computes the second-order central moments of the image region to obtain the diameters (Zaimi et al., 2018). Only tiles containing 400 or more axons were included and axons not perpendicular to the plane of the image were ignored. The dMRI was generated using the MISST toolbox (Drobnjak et al., 2010,^2011^;Ianuş et al., 2013,^2016^). Each segmented axon was represented as an infinite cylinder with diameter equal to the inner diameter of the axon. The extracellular space was represented as a tensor. FollowingWest, Kelm, Carson, Alexander, et al. (2018), the parallel diffusivity of both the cylinder and extracellular space was taken as$d_{\left|\right|}=0.35\mu^{2}/ms$and the perpendicular diffusivity of the extracellullar space was taken as$d_{⊥}=\left(1-AVF\right)d_{\left|\right|}$(H. Zhang et al., 2012), whereAVFis the axon volume fraction obtained from the segmentations. The average signalSfrom a voxel was given by:

**Fig. 1.**
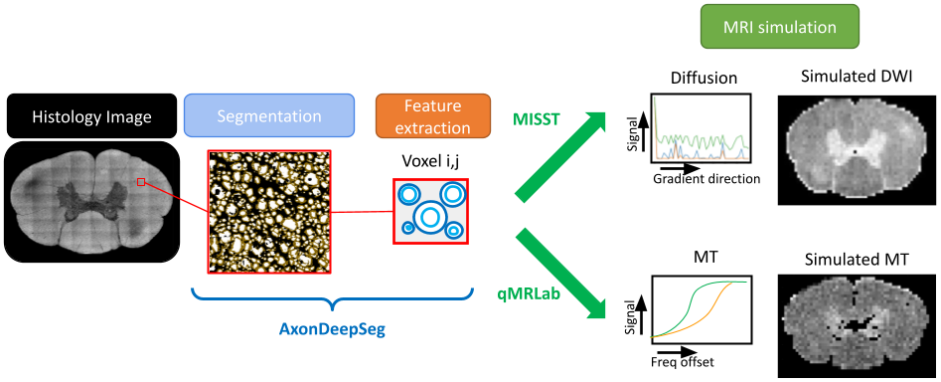
Pipeline used for generating synthetic diffusion and magnetization transfer images based on features extracted from the EM slice of a canine spinal cord. The extracted axon information is used to generate diffusion images using MISST, and the myelin content information is used to generate the MT images using qMRLab.

$$S=\left(\text{AVF}\right)\sum_{i}\frac{d_{i}^{2}}{\sum_{j}d_{j}^{2}}s_{i}+\left(\text{ECF}\right)s_{e},$$

where AVF and ECF are the axon and extracellular volume fractions obtained from the segmentation,$d_{i}$is the diameter of the i-th axon in the voxel, and$s_{i}$and$s_{e}$are the signals, obtained with MISST, coming from the i-th cylinder and the extracellular space, respectively.

The MT images were generated using qMRLab (Karakuzu et al., 2020) using the qmt_spgr module (Sled & Pike, 2000). We converted the ground-truth myelin volume fraction (MVF) to bound pool fraction using physical constants taken from the literature (West, Kelm, Carson, Alexander, et al., 2018;West, Kelm, Carson, Gochberg, et al., 2018). Namely, the volume fraction of water in myelin,$\Phi_{W,M}$, was taken as 0.475 andβ, the volume fraction of the non-myelin bound proton pool, was taken as 0.086. The protocols for the synthetic dMRI and MT images (summarized inTable 1) were chosen to match the experimental dataset described inSection 2.2.

**Table 1.** Parameters used for the experimental protocol on mice, synthetic MRI generation from dog spinal cord histology for model pretraining, and dog spinal cord MRI fromVuong et al. (2017)for evaluating the accuracy of synthetic image generation.

Sequence	TR/TE (ms)	Equivalent flip angles	Frequency offsets (Hz)	Diffusion gradient amplitudes (mT/m)	Diffusion gradient directions	_ δ _ / Δ (ms)	NEX
Synthetic image generation protocol for model pretraining							
Magnetization transfer	421 / 3.3	1000°, 4000°	1000, 2000, 4000, 6000, 10000, 22000, 30000	–	–	–	1
Diffusion	–	–	–	2 gradient amplitudes 312.7, 625.5 4 G = 0	36 directions	18 / 5	1
Synthetic image generation protocol based on Vuong et al. (2017)							
Magnetization transfer	421 / 3.3	142°, 500°	400, 800, 1000, 2200, 3000, 4000, 7000, 16000, 18000	–	–	–	1
Diffusion	–	–	–	49 amplitudes 85.7 - 600 1 G = 0	1 direction (1, 0, 0)	7/3, 25/8, 35/8, 40/8	1
Experimental protocol							
RARE (T2w)	(600, 800, 1000, 1500, 2200, 4000) / 6.3	–	–	–	–	–	1
2D FLASH - Magnetization transfer	421 / 3.3	1000°, 4000°	1000, 2000, 4000, 6000, 10000, 22000, 30000	–	–	–	1
Diffusion-weighted EPI	1050 / 56.6	–	–	2 gradient amplitudes ∼ 312.7, ∼ 625.5 4 G = 0	36 directions	18 / 5	4

Rician noise was added to all generated diffusion and MT images to obtain SNRs of 32, 100, 316, 1000 and 3160 of the White matter with respect to the background. Additionally, to evaluate the accuracy and potential biases in the synthetic images, we perform the simulation process but using the protocol in Vuong et al. (2017) in order to compare with the MRI images that are included in this public dataset. Such protocol includes the following parameters (summarized in Table 1 ):

- Magnetization Transfer (MT): Two equivalent flip angles of 142° and 500° with eight frequency offsets of 400, 800, 1000, 2200, 3000, 4000, 7000, 16000, and 18000 Hz.
- Diffusion: diffusion-weighted EPI images obtained at a single diffusion direction (1,0,0), 49 gradient amplitudes ranging from 85.7 to 600 mT/m, 1 zero gradient amplitude andΔ​/​δvalues of 7/3 ms, 25/8 ms, 35/8 ms, and 40/8 ms.

The synthetic images generated with this protocol are only used to evaluate the accuracy of the simulations and are not used for training the ML model.

### 2.2 Experimental dataset

For the transfer learning phase, we used experimental data that we previously acquired ( Chau Loo Kung et al., 2023 ). All animal procedures in that study were approved by the Stanford University Institutional Animal Care and Use Committee (IACUC) and conducted in accordance with institutional and national guidelines. Nine mouse brains were excised at 51 days after birth (P51) and were scanned using a 7T Bruker small bore animal scanner (BioSpec 70/30 USR, Gmax: 440 mT/m, SR: 3440 T/m/s) and a quadrature birdcage coil (Rapid MR International, Ohio, US). The dataset consisted of four wildtype mice, and five Scn8a ^+/^ *^mut^* mutant mice ( Makinson et al., 2017 ). The latter show generalized seizures after P21 and have been shown to have decreased g-ratio in the genu and body after seizures are well-established ( Chau Loo Kung et al., 2023 ; Knowles et al., 2022 ). The protocol parameters (summarized in Table 1 ) are as follows:

- Magnetization Transfer (MT): 2D Fast low angle shot (FLASH) images with MT pulses with equivalent flip angles of 1000° and 4000° were used with seven frequency offsets of 1000, 2000, 4000, 6000, 10000, 22000, and 30000 Hz, TE/TR = 3.3/421 ms.
- T1 images: Rapid Imaging with Refocused Echoes (RARE) images with six different TRs of 600, 800, 1000, 1500, 2200, and 4000 ms, TE = 6.25 ms.
- Diffusion: diffusion-weighted EPI images (NEX = 4) encompassed into two shells with b-values of 3000$s^{2}/mm$and 6000$s^{2}/mm$were acquired, TE/TR = 56.56/1050 ms,Δ​/​δ= 18/5 ms. Each shell consisted of 36 encoding directions with 4 additional b = 0 images.

After the MRI scan, electron microscopy images were obtained for the genu, body, and splenium of the corpus callosum. These regions are indicated on the structural mouse brain scan shown inFigure 2. We quantified 7–14 separate electron micrographs for each of the three callosal regions in each mouse. In this way, approximately 200 axons were depicted for each region of interest in each mouse. The diameter of each axon was divided by the diameter of each axon and its myelin sheath along the short axis of axonal cross-sections to determine a g-ratio as described in previous literature (Geraghty et al., 2019;E. M. Gibson et al., 2014;Knowles et al., 2022;Smith & Koles, 1970).

**Fig. 2.**
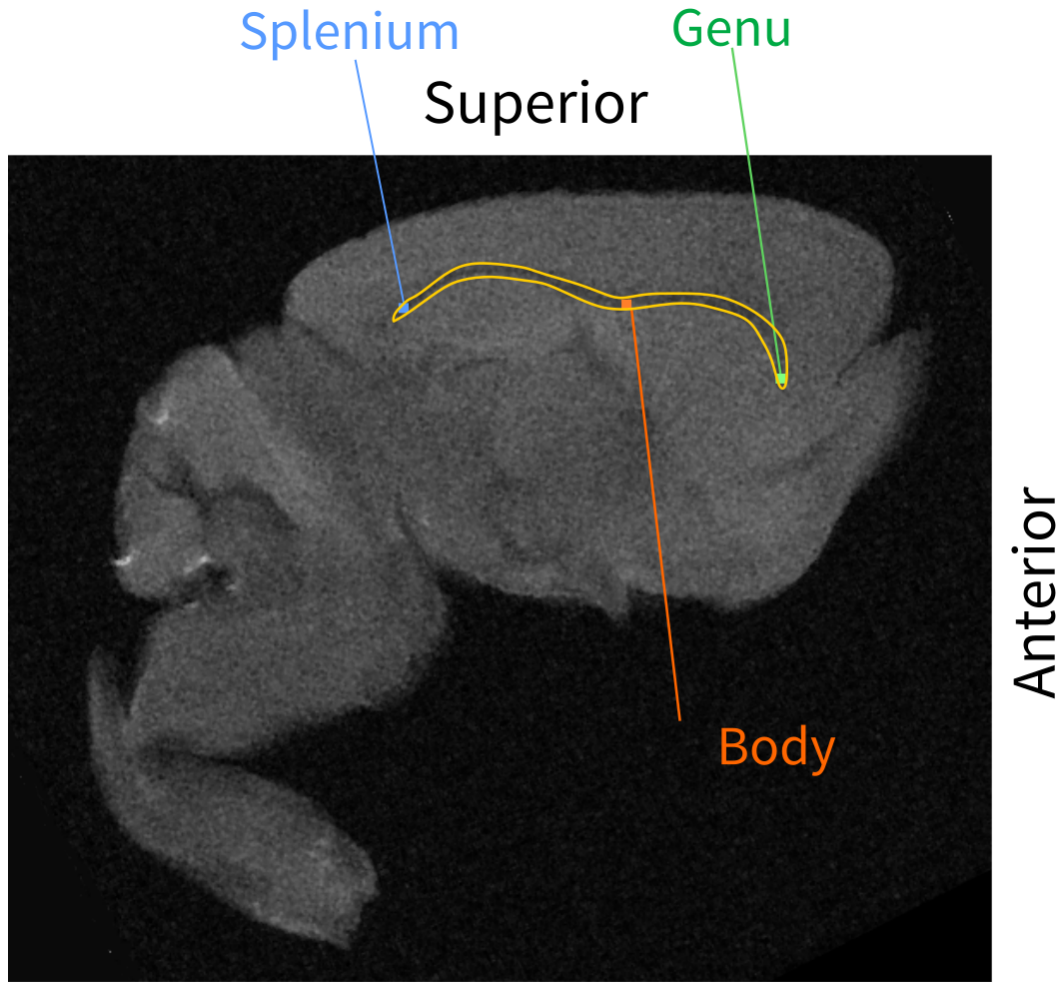
Structural MRI of an*ex vivo*mouse brains. The corpus callosum is outlined in yellow and representative regions corresponding to the genu, body, and splenium are labelled.

### 2.3 Deep learning architecture

**Fig. 3.**
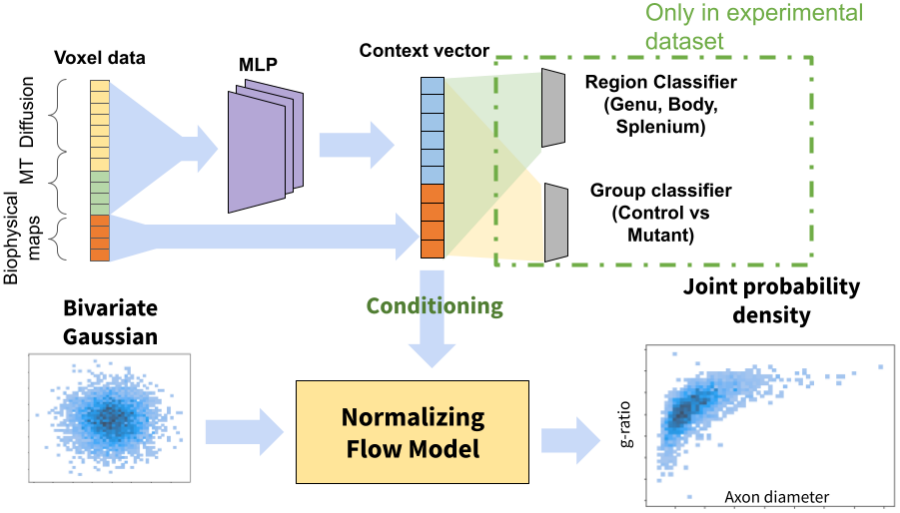
Architecture of the network to predict joint axon diameter and g-ratio distributions. The MT and diffusion values of a voxel are passed through a multi-layer perceptron (MLP) encoder and concatenated with the information from the biophysical maps to form a context vector. The context vector is used to condition a normalizing flow model that learns an invertible transformation from a bivariate Gaussian to the joint distribution of axon diameters and g-ratios. Only during transfer learning, classification heads are added to the context vector to train the network in a multi-task fashion.

Inspired byJallais and Palombo (2023), we used a conditional Normalizing Flow Model (cNFM) (Cramer et al., 2023;Rezende & Mohamed, 2015) to achieve non-parametric prediction of axon diameter and g-ratio distributions from the Diffusion and MT values of an MRI voxel as well as biophysical maps that are fitted from these. The cNFM consists of a series of invertible transformation blocks that map a known basic distribution (e.g., bivariate Gaussian) to the target transformation, conditioned by a context vector (Fig. 3). We chose to use two masked autoregressive blocks (Papamakarios et al., 2017). The architecture is shown inFigure 3. The context vector is generated using a multi-layer perceptron (MLP) which encodes the concatenated diffusion and MT values of a voxel into a 16-dimensional vector. This MLP encoder consists of three layers of 64, 32, and 16 units with a Parametric ReLU (PReLU) activation function. A batch normalization layer is included before the second and third layers.

To make the network more physically-informed, we include the outputs of three biophysical models that are concatenated to the context vector before conditioning the NFM. These maps include outputs from the neurite orientation dispersion and density imaging (NODDI) model (H. Zhang et al., 2012) (intra-cellular volume fraction, isotropic volume fraction, orientation dispersion), outputs from the diffusion tensor imaging (DTI) model (fractional anisotropy, mean diffusivity) and from fitting the MT curves (Karakuzu et al., 2020) (Exchange rate and bound pool fraction). All of these models are fitted voxel wise. We decided to use the NODDI model as it has been extensively used as an intermediate step for g-ratio modeling (Lu et al., 2024;Stikov et al., 2015;West, Kelm, Carson, Alexander, et al., 2018) and as a compromise between scan time and accuracy. Some of the limitations of NODDI with respect to T2 variations in the compartments and the effects on g-ratio have been investigated before (Chau Loo Kung et al., 2023). More advanced models such as the soma and neurite imaging by diffusion (SANDI) (Palombo et al., 2020) model or multi-TE NODDI (Gong et al., 2020) could be considered at the cost of additional diffusion shells or repetitions with different TEs, respectively.

Additionally, we discovered that using classification heads attached to the context vector to classify the region (genu, body or splenium) and the mouse group (Scn8a^+/+^or Scn8a^+/^*^mut^*) improved the overall performance of the network. The network was implemented in PyTorch and for the NFM we used the implementation provided byStimper et al. (2023). Code and trained models are available inhttps://github.com/gustavochau/Brain_microstructure_initial.

### 2.4 Pretraining on synthetic data

The ML model was first trained on synthetic data, and this step will be referred to as pretraining. The objective is for the network to first learn some useful general features in the encoder that can later be translated to the experimental domain. This step is performed to partially compensate for the low amount of data in the final target experimental dataset. Pretraining was performed in 1600 voxels, corresponding to 80% of the synthetic data, using a negative log-likelihood (NLL) loss. The loss is given by:

$$\text{NLL}=-\sum_{d,g,\text{x}\in \left\{D,G,X\right\}}\text{log}\;\;{\left(P(d,g|\text{x})\right)}_{\theta },$$

where the sum is over all the axon diameter and g-ratio samples,dandgrespectively on the training dataset samplesDandG, and the corresponding MRI values vectorxof the given voxel.$P{\left(d,g|\text{x}\right)}_{\theta }$is the probability predicted by the network with parametersθconditioned on the MRI vectorxand evaluated for axon diameterdand g-ratiog.

Multiple models were trained with and Adam optimizer and different hyperparameters using wandb ( Biewald, 2020 ), and the best model was chosen based on the Jensen-Shannon distance (JSD) between the predicted distribution and the histogram of the ground-truth samples on the validation set. The JSD measures the similarity between two probability distributions and is the average divergence of the two distributions from their mean. The explored hyperparameters included:

- The number of blocks and hidden units in the NFM.
- Dropout rate for the encoder.
- Number of epochs.
- Learning rate (Uniformly sampled between 1E-4 and 1E-3).
- Weight decay (Uniformly sampled between 1E-6 and 1E-5).

### 2.5 Transfer learning on experimental data

For the transfer learning stage, each sample consists of the concatenated MRI values of the centroid of each corpus callosum ROIs and the list of EM axon diameters and g-ratios. The ROIs were manually drawn for each of the regions. We performed a 5-fold cross-validation. Five permutations of the nine mice in training/validation/test were selected from the 120 valid permutations consisting of two Scn8a ^+/+^ and three Scn8a ^+/^ *^mut^* mice for training, one of each class for validation, and the same number for test. We considered a permutation valid if the mouse whose splenium EM was missing due to experimental issues was included in the training set. For each fold, we fine-tuned the pretrained network using different hyperparameters sweeps, including different layer freezing strategies. As previously mentioned, for the fine tuning in the experimental data, we added two heads for classifying the region and the group, and trained in a multi-task fashion by alternating between a cross-entropy loss for classification and negative log-likelihood for the NFM. The best model was chosen on the validation set based on two metrics of the g-ratio distribution:

- The percentage error in the mean of the predicted distribution of g-ratios by the network versus the mean of the EM ground truth. That is,
  $$\text{\% Error}=\frac{\left|\mu_{EM}-E\left[g\right]\right|}{\mu_{EM}},$$
- where$\mu_{EM}$is the sample mean obtained from the EM g-ratio values andE[g]is the expected value of g-ratio under the probability distribution predicted by the model.
- The effect size of the genu, body and splenium calculated as the Cohen’s d between the Scn8a^+/+^and Scn8a^+/^*^mut^*mice. The effect size takes into account both training and validation data.

### 2.6 Evaluation and comparison with biophysical modeling

The “point metrics” defined in the previous section (percentage error and effect size) allow a comparison with a conventional biophysical model approach in the g-ratio prediction task. This biophysical model provides a single aggregated g-ratio estimate in a given voxel as:

$$g_{MRI}=\sqrt{\frac{1}{1+\frac{MVF}{AVF}}},$$

where MVF is the myelin volume fraction, which is estimated from the MT images, and AVF is the axon volume fraction obtained from the diffusion sequence (Stikov et al., 2015;West, Kelm, Carson, Alexander, et al., 2018;West, Kelm, Carson, Gochberg, et al., 2018). The details of the fitting of the model are provided inChau Loo Kung et al. (2023).

For both validation and testing, we took the inference of the network for each of the individual voxels of each ROI (each one is a joint probability distribution) and averaged all of these together to obtain a final predicted distribution for the ROI. Finally, the model selected in validation for each fold was evaluated on the test set for that particular fold and we report the average and standard deviations obtained from the five evaluated folds. For comparing the whole distribution, we report the Jensen-Shannon distance and the Kullback–Leibler divergence (KLD) between the distribution predicted by the model and the EM ground-truth histogram. For reference, we report these same metrics but compare a direct fitting of the EM samples using a Gamma distribution and the EM ground-truth histogram. We selected a Gamma distribution as the reference as it has been used in previous studies for both axon diameter and g-ratio (Assaf et al., 2008;West et al., 2016).

### 2.7 Additional experiments

To delve deeper into the overall performance of the approach, we performed an ablation experiment in which certain parts of the ML model are removed to investigate the dependence on each of them. The models considered for ablation are:

1. Only the diffusion input and diffusion derived maps as inputs.
2. Only the MT input and MT derived maps as inputs.
3. Without using the biophysical maps as additional inputs.
4. With the classification heads removed (No multi-task approach)
5. Skipping pretraining and transfer learning, and training directly on the experimental mouse data.
6. Pre-training with less samples (50% and 75% of the synthetic data).

To evaluate the generalization of the trained model, we also performed further using additional data:

1. We tested the model on a different group of brains scanned at P21 (4 Scn8a^+/+^and 5 Scn8a^+/^*^mut^*) following the same protocol described inSection 2.2. These mice do not have paired EM ground-truth but the general trends in g-ratio values can be estimated using previous biological studies (Knowles et al., 2022). We compared the model’s prediction all along the corpus callosum besides the three regions that were considered in the training/validation/test splits. Please note that for P21 mice some erosion to the masks was performed to avoid some low g-ratio values in the borders between the corpus callosum and surrounding tissue.
2. We tested expected distribution shifts obtained from sampling from the predicted distributions in the voxels of the callosal body that were not included in any of the splits and compared with previous studies (Knowles et al., 2022).

## 3 Results

### 3.1 Synthetic images

**Fig. 4.**
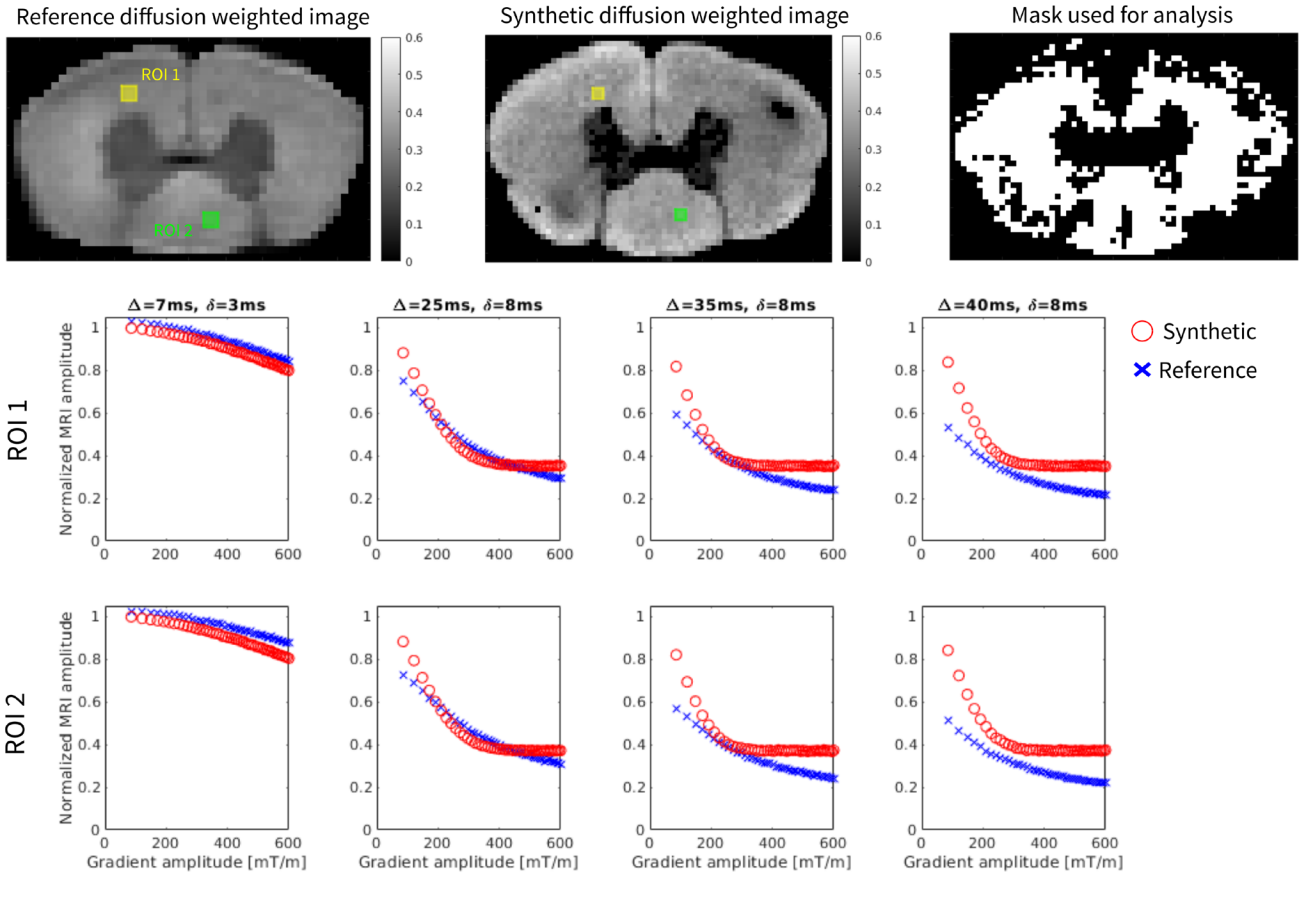
Comparison of the reference and synthetic diffusion-weighted images with aΔ=25 ms,δ= 8 ms, and|G|= 600 mT/m. The yellow and green squares indicate the ROIs used for the comparison plots shown in the bottom. The normalized diffusion values are presented in red for the synthetic images and in blue for the reference image, across different combinations ofΔ,δand|G|. In the top right corner, a mask of the selected voxels used for evaluating the synthetic images and training the model is also included.

Examples of generated synthetic diffusion-weighted MRI images generated with the protocol inVuong et al. (2017)are shown inFigure 4. Reference MR images that were included inVuong et al. (2017)are also shown for comparison. The simulations are only run for pixels inside white matter, represented by the mask inFigure 4. A quantitative comparison between the synthetic and experimental images for diffusion and MT contrasts across different protocols is summarized inTable 2. Additionally, for training the ML model and analysis, we only consider voxels whose segmentation had more than 400 axons (such mask is shown inFig. 4).Figure 4shows that the synthetic values tend to align with the experimental one for lower values of mixing timeΔ= 7 and 25 ms. At higher mixing times and especially at higher gradient values, the simulations seem to saturate. This could be attributed to additional and smaller tissue structures which are captured by the stronger diffusion gradients but are not being taken into account by the simplified model used in the simulations. We note thatΔvalues used in the actual protocol for the pretraining is 18 ms, which would be in the range where there is still a good agreement of the curves. Comparisons for the MT (magnetization transfer) simulations are provided inFigure 5. In these comparisons, the synthetic ROIs exhibit trends similar to the experimental values for both flip angles, although there is a positive bias in the normalized values, particularly at lower frequency offsets.

**Table 2.** Mean±standard deviations of the differences for the various statistics comparing the synthetic images and the reference MRI images.

	Difference in means	Difference in median	Difference in standard deviations
Diffusion, Δ = 7 ms, δ = 3 ms	-0.029 ± 0.004	-0.041 ± 0.009	-0.054 ± 0.016
Diffusion, Δ = 25 ms, δ = 8 ms	-0.005 ± 0.042	-0.014 ± 0.043	-0.002 ± 0.016
Diffusion, Δ = 35 ms, δ =8 ms	0.053 ± 0.045	0.048 ± 0.046	0.014 ± 0.012
Diffusion, Δ = 40 ms, δ = 8 ms	0.091 ± 0.047	0.087 ± 0.047	0.019 ± 0.013
MT, angle = 142°	0.105 ± 0.092	0.112 ± 0.093	-0.002 ± 0.010
MT, angle = 500°	0.181 ± 0.219	0.192 ± 0.226	0.007 ± 0.038

**Fig. 5.**
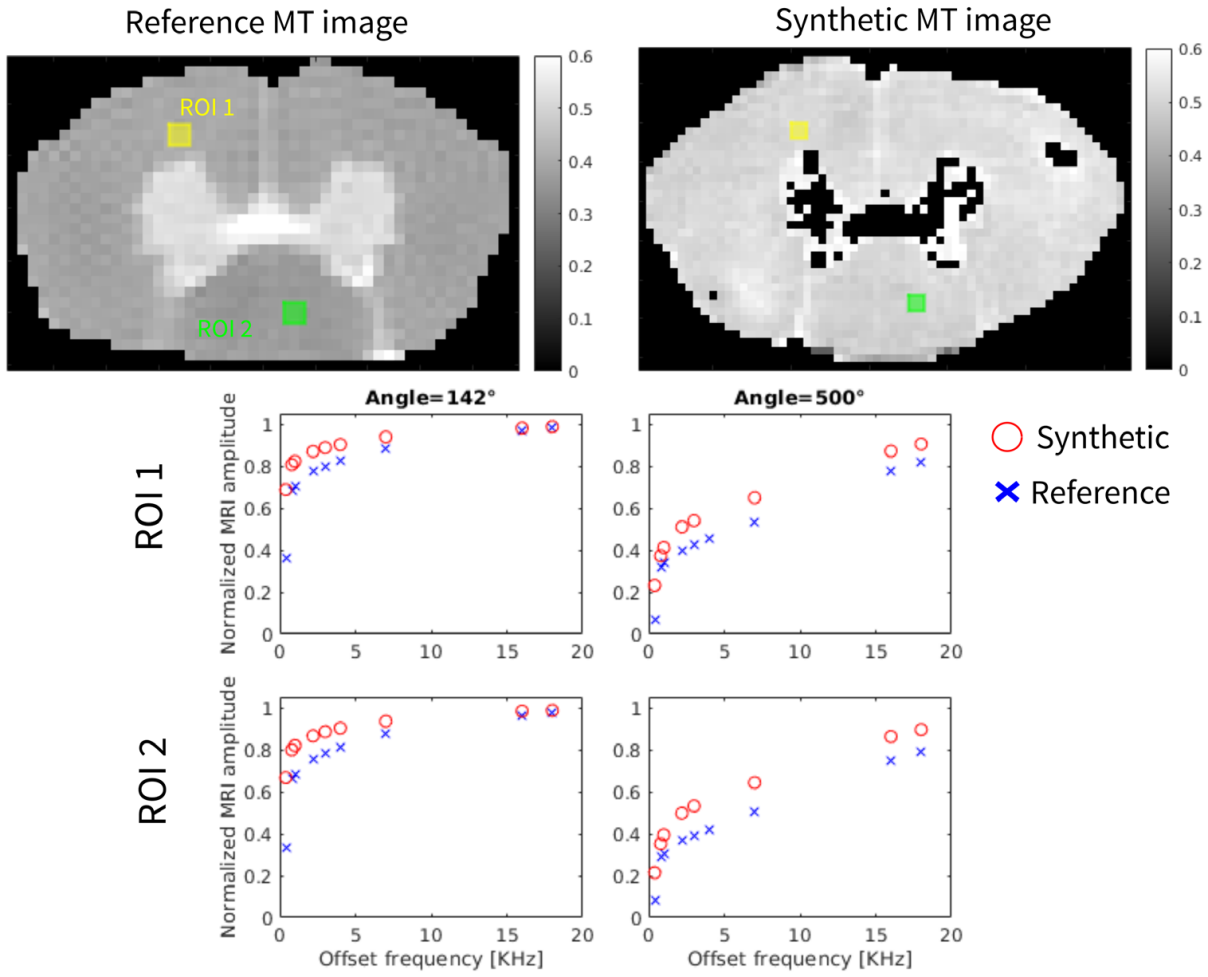
Comparison of the reference and synthetic MT images with an equivalent flip angle of 500° and offset frequency of 2.2 KHz. The yellow and green squares indicate the ROIs used for the comparison plots show in the bottom. The values of the normalized MT values are shown in red for the synthetic images and blue for the reference image, for the different combinations of equivalent flip angles and frequency offsets.

### 3.2 Pretraining

**Fig. 6.**
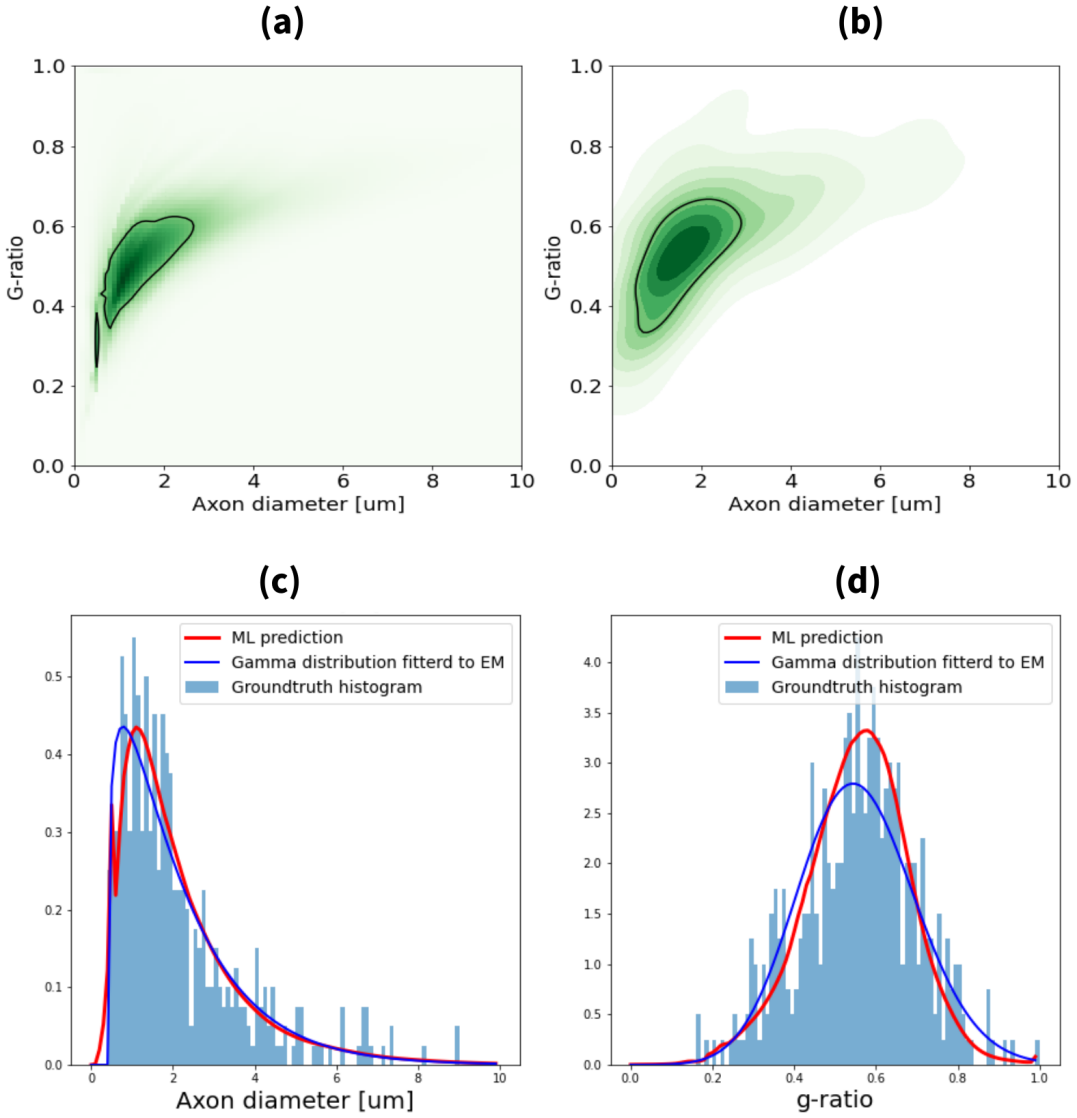
Results showing the prediction of the pretrained network on synthetic data. (a) shows a kernel density estimation (KDE) plot of the samples for a particular voxel and (b) the network predicted distribution for the same voxel. The marginal distributions predicted by the model are shown in red for axon diameter (c) and g-ratio (d) with the ground-truth histograms in light blue. A gamma distribution fitted to the ground truth is shown in dark blue.

An example of the joint distribution and marginal distributions predicted by the model in a voxel of the test set is shown inFigure 6.Figure 6shows an example of the joint and marginal distributions predicted by the model for a voxel in the test set. Overall, there is good visual alignment between the model’s predicted distribution and the ground-truth distribution, represented by the kernel density estimation (KDE) plot. This alignment is evident in both the range (support) and the central tendency (centroid) of the joint distributions. The marginal distributions also closely match the model’s predictions. For comparison, a gamma distribution fitted directly to the ground-truth data (EM) is displayed in dark blue. Across the test set, the mean Jensen-Shannon divergence (JSD) values for g-ratio and axon diameter were 0.18 and 0.18, respectively.

### 3.3 Results of transfer learning in experimental dataset

**Fig. 7.**
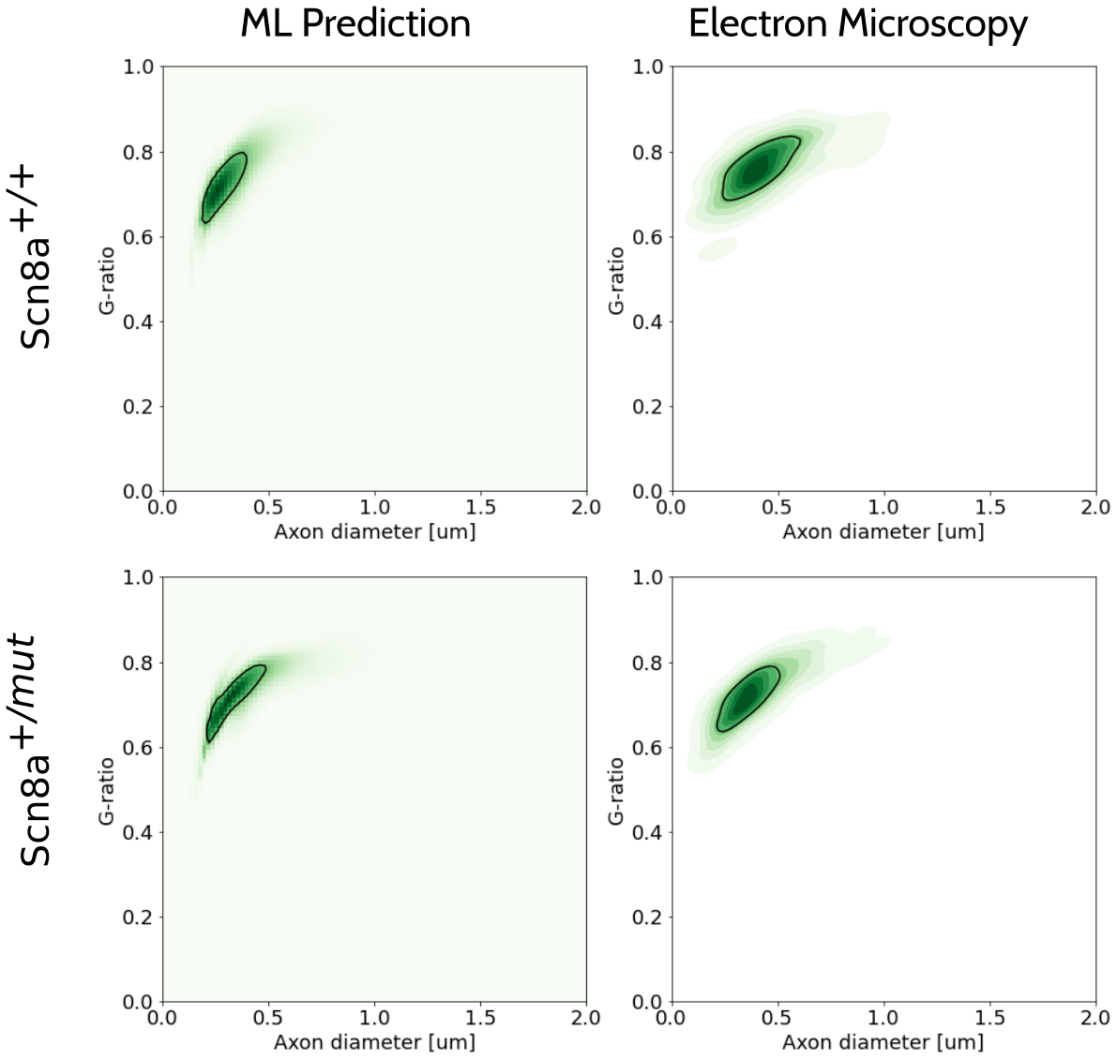
Comparison of the joint distribution predicted by the network after transfer learning for both the Scn8a^+/+^mouse and the Scn8a^+/^*^mut^*mouse. KDE plots of the electron microscopy samples are also shown for comparison. Black contours corresponding to half the maximum value of the distributions are shown.

**Fig. 8.**
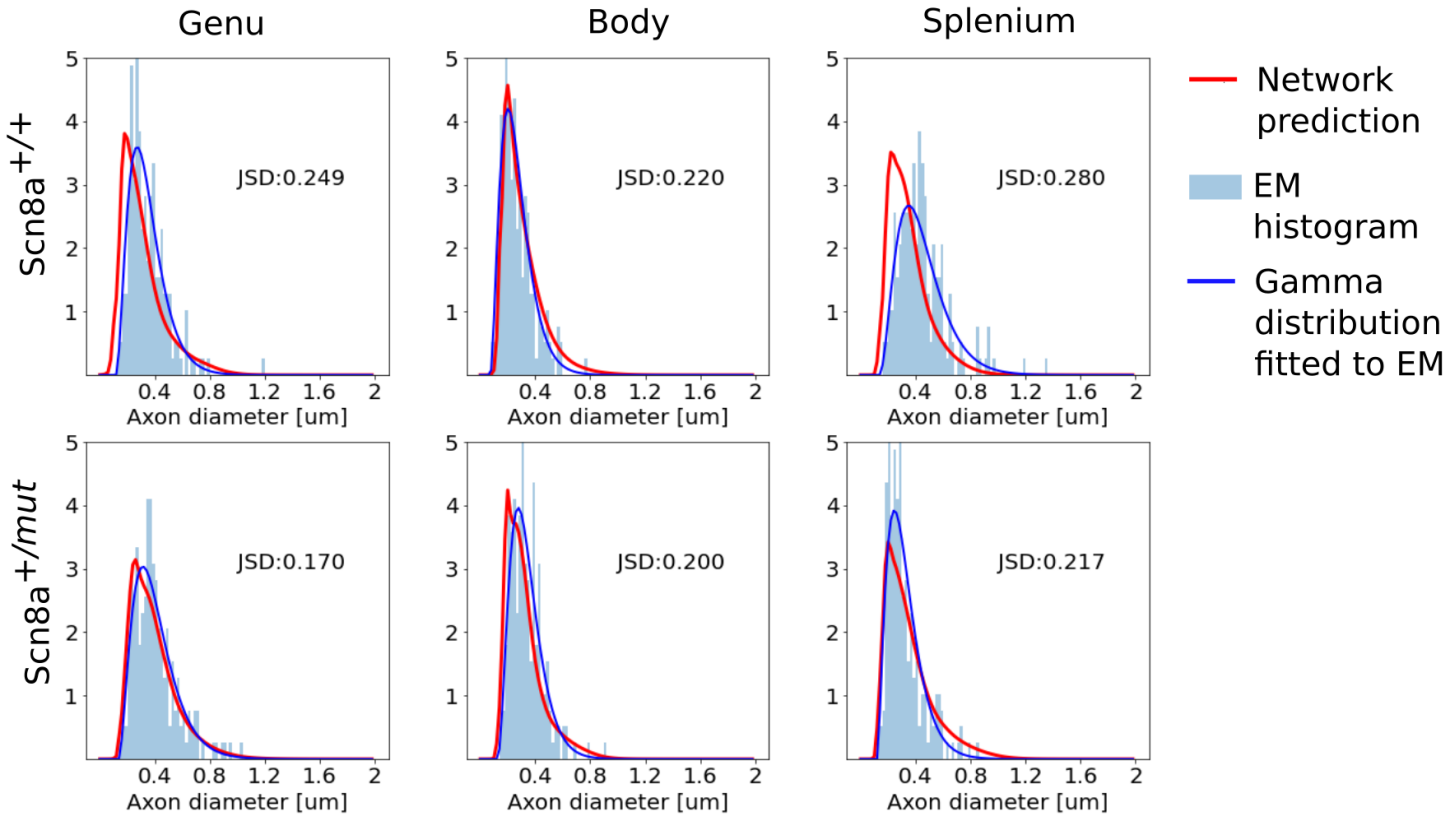
Plots showing the histogram of samples (light blue) and predicted distribution from the network (continuous red line) for the axon diameter prediction for the Scn8a^+/+^and Scn8a^+/^*^mut^*mice in the test set. Direct fitting of EM ground truth with a Gamma distribution is shown in solid dark blue.

Figure 7shows a comparison of the joint distribution predicted by the network after transfer learning with a kernel density estimate plot of the samples for the callosal body of the Scn8a^+/+^and Scn8a^+/^*^mut^*mouse of the test set. A good visual agreement can be observed in the centroid and support of the distributions. The marginal distributions for all the samples in the test set are shown inFigures 8and9for axon diameter and g-ratio, respectively. Overall, we see a high correspondence between the network prediction and the ground truth. However, the predictions of axon diameter do present some wider spreads or offsets in some cases (e.g.Fig. 8, genu or splenium of Scn8a^+/+^orFig. 8, splenium of Scn8a^+/+^). As a reference,Figures 8and9also show a Gamma distribution directly fitted to the EM groundtruth, which in reality is not feasible with MRI alone.

**Fig. 9.**
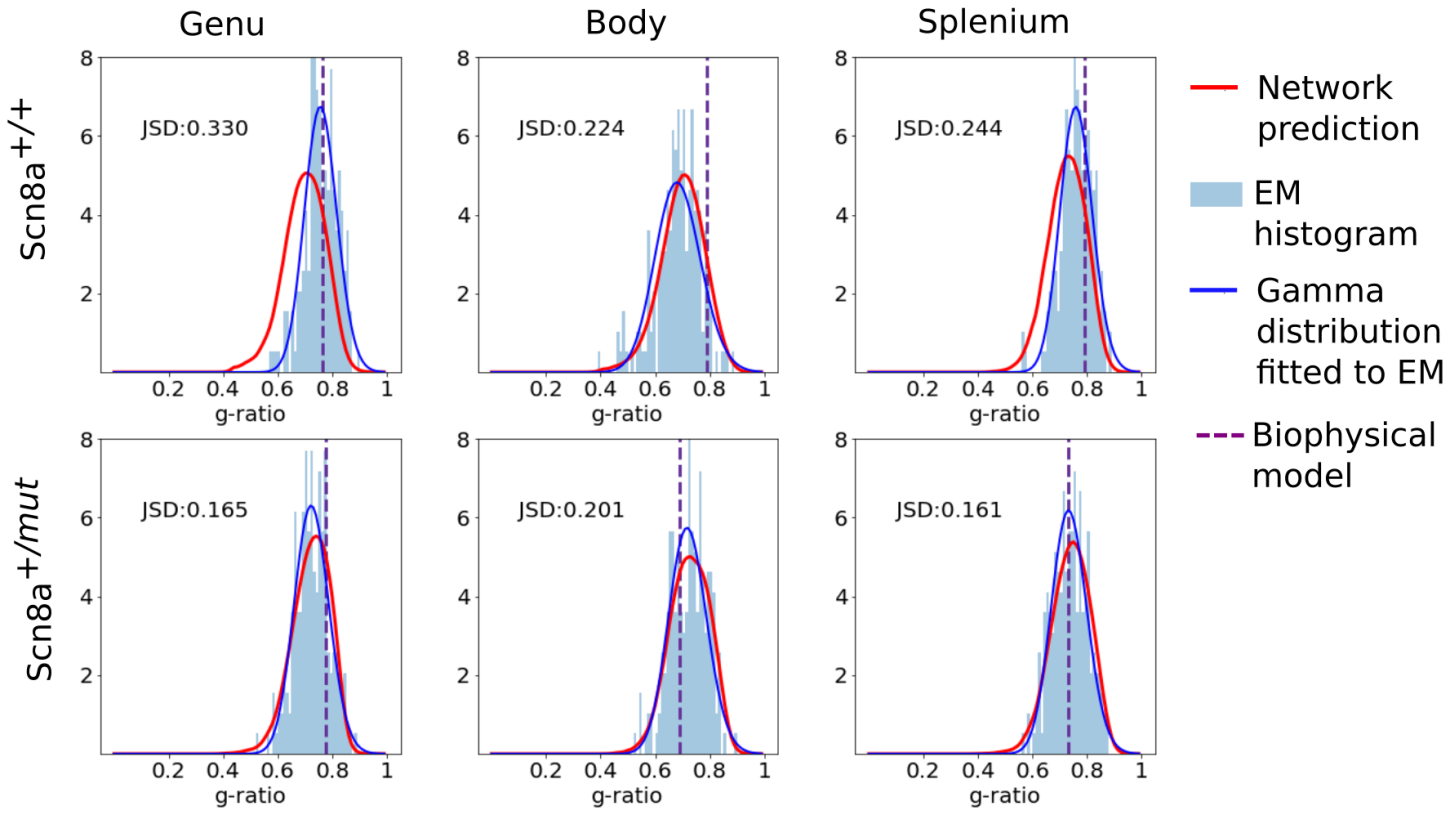
Plots showing the histogram of samples (light blue) and predicted distribution from the network (continuous red line) for the axon diameter prediction for the Scn8a**^+/+^**and Scn8a**^+/^***^mut^*mice in the test set. The predictions of the volume fraction model for aggregated g-ratio are shown as a purple vertical line. Direct fitting of EM ground truth with a Gamma distribution is shown in solid dark blue.

We show the performance metrics on the test set inTable 3for point metrics. We observe a reduction of around 3.5% in the mean errors of the genu and body in the ML prediction compared to the biophysical model. Although 3.5% might appear a small improvement, at normal g-ratio values, for normal values of g-ratio in the corpus callosum this can be equivalent to approximately 0.02–0.03 which is in the order of magnitude of the changes we might want to detect in certain pathologies (Chau Loo Kung et al., 2023;Knowles et al., 2022). We observe also an effect size closer to the EM effect size in both the genu and body, although not in the splenium, but we note that these group effects take into account the training and validation sets.

**Table 3.** Performance of the ML model compared with biophysical model and EM groundtruth in terms of mean values and group metrics.

	Method		
Metric	EM groundtruth	Biophysical	ML
Genu (% error in mean)	-	7.83% ± 3.53%	4.32% ± 4.86%
Body (% error in mean)	-	6.09% ± 2.10%	2.79% ± 2.73%
Splenium (% error in mean)	-	3.06% ± 1.41%	3.02% ± 2.30%
Genu effect size	4.01	1.60	1.76 ± 1.23
Body effect size	2.44	3.71	3.02 ± 3.19
Splenium effect size	-0.45	-0.26	-0.39 ± -0.29

We show the performance metrics on the test set inTable 4for distribution-wide metrics. The distances to a Gamma distribution fitted to the EM ground truth are also shown for reference. The axon diameter distances are higher than a direct fitting, but for g-ratio, we observe that the JSD and KLD are quite close to a direct fitting of the distribution.

**Table 4.** Performance of the ML model in terms of distribution-wide metrics.

	Method	
Metric	ML prediction	Direct fitting
JSD axon diameter	0.24 ± 0.25	0.18 ± 0.01
KLD axon diameter	1.42 ± 1.53	0.63 ± 0.12
JSD g-ratio	0.23 ± 0.23	0.18 ± 0.01
KLD g-ratio	1.32 ± 1.38	0.80 ± 0.06

A comparison to a direct fitting of a gamma distribution to the EM samples is included for reference.

### 3.4 Additional evaluations

Table 5shows the result of the ablation analysis for both point metrics and distribution-wide metrics. We observe that although some metrics are slightly better for the no-pretrain version, overall the pretraining helps maintain a lower error in the g-ratio means and lower JSD and KLD. Decreasing the number of samples for pretraining also impacts the overall performance. In a similar way, the model without the multi-task shows higher overall percent errors in some regions, and without the additional classification task, the effect size is not that well maintained as the model is not encouraged to differentiate between groups. We also notice that the inclusion of the biophysical maps is necessary to obtain a better performance. Finally, the models that only use the diffusion data or the MT data alone do not perform as well (e.g., effect size in the genu for diffusion only and effect size in the body for MT only), suggesting that we indeed need the information coming from the multi-modal approach.

**Table 5.** Comparison of the different models considered in the ablation experiments.

	Model							
Metric	Baseline	No biophysical maps	No multitask	No pretraining	Diffusion only	MT only	50% samples in pretraining	75% samples in pretraining
Genu (% error in mean)	4.32% ± 4.86%	4.56% ± 4.67%	4.77% ± 5.14%	4.77% ± 4.97%	4.33% ± 4.50%	4.59% ± 4.61%	4.65% ± 4.83%	6.30% ± 6.91%
Body (% error in mean)	2.79% ± 2.73%	2.55% ± 2.17%	3.40% ± 3.39%	2.41% ± 2.30%	2.08% ± 1.78%	2.66% ± 2.66%	2.59% ± 2.38%	3.07% ± 2.77%
Splenium (% error in mean)	3.02% ± 2.30%	2.56% ± 2.04%	2.33% ± 1.91%	3.37% ± 2.97%	2.64% ± 2.26%	3.28% ± 2.69%	3.25% ± 2.98%	4.59% ± 3.87%
Genu effect size	1.76 ± 1.23	1.32 ± 1.30	1.13 ± 0.88	1.53 ± 1.39	1.09 ± 1.06	1.83 ± 1.61	1.41 ± 1.00	0.62 ± 0.45
Body effect size	3.02 ± 3.19	1.57 ± 1.65	2.86 ± 3.05	2.08 ± 1.96	2.70 ± 2.77	0.91 ± 0.61	1.96 ± 2.06	1.98 ± 2.20
Splenium effect size	-0.39 ± -0.29	-0.48 ± -0.38	-0.49 ± -0.47	-0.09 ± -0.06	0.17 ± 0.33	1.06 ± 1.42	0.14 ± 0.18	-0.12 ± -0.05
JSD g-ratio	0.24 ± 0.25	0.23 ± 0.23	0.23 ± 0.23	0.28 ± 0.28	0.24 ± 0.24	0.22 ± 0.22	0.24 ± 0.23	0.29 ± 0.30
KLD g-ratio	1.42 ± 1.53	1.20 ± 1.20	0.96 ± 0.95	2.07 ± 2.11	1.13 ± 1.17	0.78 ± 0.78	0.96 ± 0.96	2.18 ± 2.39
JSD axon diameter	0.23 ± 0.23	0.23 ± 0.23	0.23 ± 0.23	0.26 ± 0.26	0.22 ± 0.21	0.23 ± 0.23	0.23 ± 0.23	0.28 ± 0.28
KLD axon diameter	1.32 ± 1.38	1.28 ± 1.35	1.28 ± 1.30	1.84 ± 2.00	0.85 ± 0.89	1.24 ± 1.29	1.14 ± 1.21	1.75 ± 1.91

To assess the potential bias introduced by pretraining the model on larger axon diameters before applying transfer learning to smaller ones, we conducted additional simulations. The axon diameter distribution from the dog spinal cord was artificially scaled down by a factor of 0.15 to align its mean with the experimental mouse data, reducing the mean diameter from approximately 2.07μm to 0.31μm. The myelin volume fraction was kept constant during the MT simulations. The model was then pretrained on this modified synthetic dataset before transfer learning was applied to the mouse experimental data. Results, summarized inAppendix, show that pretraining on synthetic images derived from smaller axons yielded a model performance (i.e., % error in mean in the test set) of 3.80%±3.63% across all brain regions). In comparison, the baseline model (pretrained on synthetic data from the dog spinal cord) achieved a performance of 3.38%±3.30%. These observations indicate that the axonal size discrepancy between the pretraining dataset and the experimental dataset does not significantly impact the final model’s performance.

**Fig. 10.**
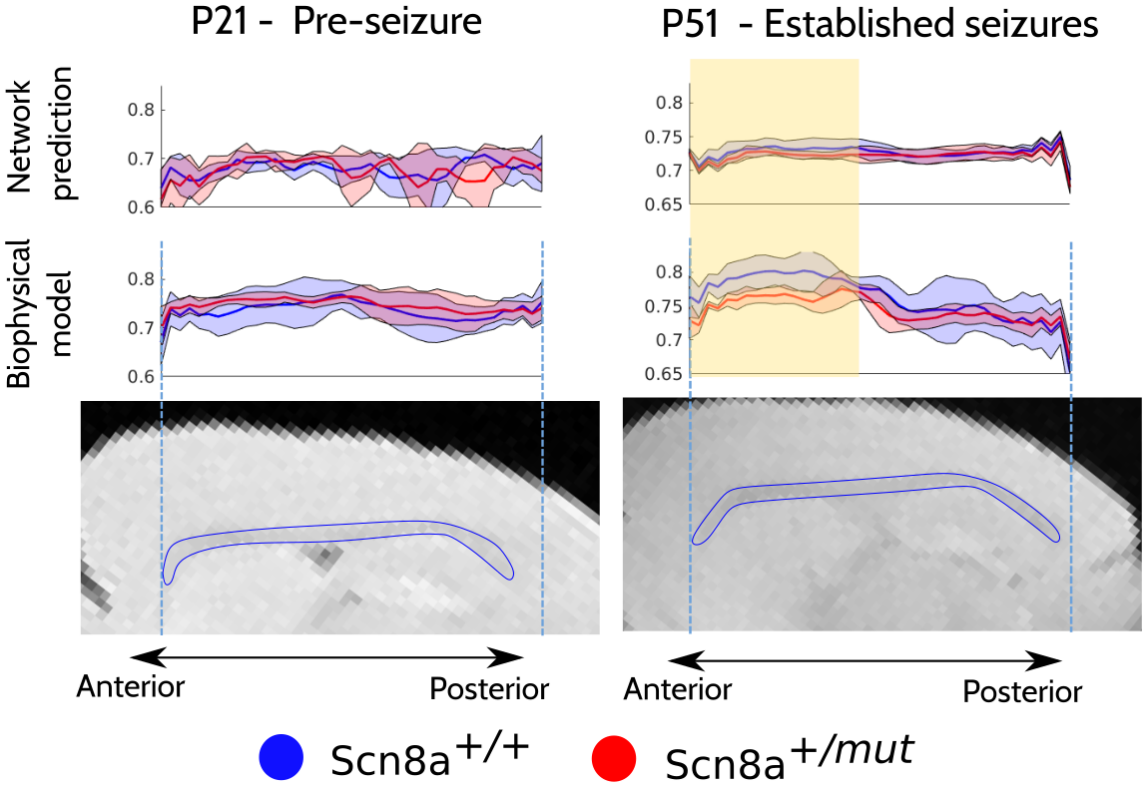
Group means and standard deviation curves of MRI measurements of g-ratio along the anterior-posterior extent of midline corpus callosum before seizures (P21) and after established seizures (P51). Those obtained from the network prediction are shown at the top row, while those from the biophysical model are shown in the center row. Representative brain slices are shown for spatial reference with the corpus callosum outlined in blue. The yellow highlight indicates the approximate anterior region where the g-ratio of Scn8a^+/^*^mut^*mice is lower. The P51 brain corresponds to an Scn8a^+/+^mouse, and the P21 corresponds to an Scn8a^+/^*^mut^*mouse.

Figure 10depicts mean g-ratios measured continuously in the corpus callosum along the anterior-posterior axis. Mean g-ratios were taken from corpus callosum regions of interest that were manually delineated in several slices, excluding the two most lateral ones at each hemisphere. The model prediction is shown on top while the biophysical model prediction is shown in the middle row. None of the voxels used in the training/validation/test splits are used for this analysis. In both cases, we observe that mean Scn8a^+/^*^mut^*(red) g-ratio curves overlap with the Scn8a^+/+^for mice without seizures (at P21), whereas Scn8a^+/^*^mut^*g-ratios are lower than Scn8a^+/+^between the genu and the body (highlighted in yellow) for mice with established seizures (at P51). This is clearer for the biophysical model but there is still an observable difference in the ML predictions despite the variance. We note that the curves are generated with all mice available (including training and validation) but do not include any of the voxels used in the training/validation/test splits. A spline regression analysis shows that the ML-predicted curves are significantly different between the two groups for P51 (p-value=0.0027) but not for P21 (p-value=0.88).

**Fig. 11.**
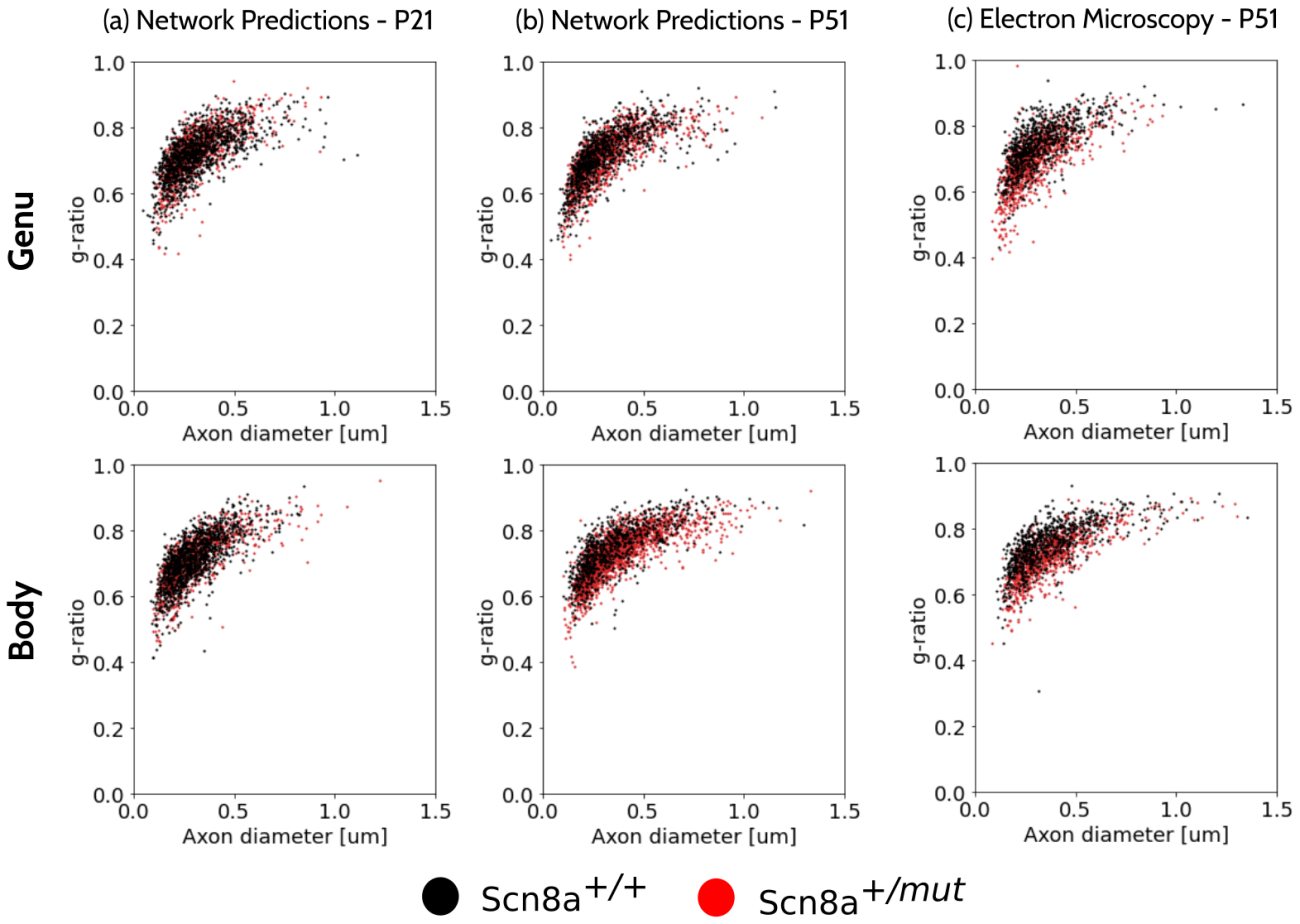
2D scatter plots obtained from the EM of P51 mice (c) and from sampling from the trained ML model for P21 (a) and P51 (b), for the genu (top row) and body (bottom row). Scn8a^+/+^mice are shown in black and Scn8a^+/^*^mut^*mice are shown in red. A downward shift of the red cloud is observed at P51 for the network prediction and the EM ground truth, consistent with the expected reduction of g-ratio but not axon diameter from this and previous studies (Knowles et al., 2022).

Figure 11shows scatter plots obtained from sampling the joint distributions predicted by the model for collosal body voxels at P21 (Fig. 11a) and P51 (Fig. 11b). The sampling comes from voxels from every one of the nine mice besides the centroid of the ROIs. For reference, we also show the scatter plot of the EM values for P51 mice (Fig. 11c). We observe a downward shift in the red point cloud, corresponding to Scn8a^+/^*^mut^*mice in P51 as expected from the change in g-ratio but not in axon diameter. The plots obtained from the model closely follow those presented in previous biological studies (Knowles et al., 2022).

## 4 Discussion

We have presented a novel approach for non-parametric prediction of joint-distributions of microstructural features. Previous studies using ML to predict brain microstructure have used previous biophysical model fitting outputs as the ground truth (Lim et al., 2022;Ye et al., 2019), or have used publicly available histological images as the register to their acquired MRI on different as ground truth (Liang et al., 2022,^2023^). To our knowledge, this is the first study that has trained an ML model on paired MRI/histology data that were acquired on the same samples for the purpose of predicting microstructural features directly from the MRI data. Our approach seeks to circumvent the challenge of obtaining high volumes of paired MRI/histological data by pretraining on synthetic MRI images and then performing transfer learning. Additionally, multi-task learning techniques were used to stabilize training. We consider this a general approach, but in this study, it was used to predict distributions of g-ratio and axon diameters. We expect that the use of a non-parametric NFM can provide advantages to translate to new applications or other white matter areas.

Our comparisons in a mouse brain experimental dataset showed a good agreement between the distributions predicted by the network and those obtained by directly fitting the histological samples, alongside reduced mean error compared to a pure biophysical approach. Quantitative evaluations showed Jensen-Shannon Divergences of 0.28 and 0.21 in axon diameter and g-ratio, respectively, compared to direct fitting values of 0.18 in both scenarios. The results of the ablation experiments suggest that the approach of pretraining with synthetic data can help alleviate the lack of big datasets containing both MRI and histological data. In our case, adding an additional auxiliary correlated task and training in a multi-task fashion stabilized the training and helped performance. Indeed, there is previous evidence that correlated auxiliary tasks can improve the performance of the model in the primary task (Liebel & Körner, 2018).

Our additional experiments (Section 2.7) show that the model is able to generalize to the mouse corpus callosum as a whole as well as data acquired at a different developmental stage (Fig. 10). In addition,Figure 11is a good example of how the trained model is able to generate predictions that are consistent with previous biological priors (Knowles et al., 2022). However, the question of generalization to other brain regions (and in particular to gray matter) still remains open.

Despite these encouraging results, several limitations of our approach should be acknowledged. First, the inherent sensitivity limits of MRI must be considered. Previous studies have highlighted that diffusion MRI loses sensitivity for smaller axon diameters, where the signal becomes increasingly indistinguishable from noise, particularly beyond the inherent resolution limit. This phenomenon leads to an under-representation of small axons in the diffusion MRI signal (Drobnjak et al., 2016;Nilsson et al., 2017).Nilsson et al. (2017)provides a theoretical analytical approximation of the minimum detectable axon diameter$d_{min}$for single diffusion encoding acquisition:

$$d_{min}={\left(\frac{768}{7}\frac{\sigma D_{0}}{\gamma_{h}^{2}\delta g_{exp}^{2}}\right)}^{1/4}$$

whereσis the effective standard deviation of the signal due to noise,$D_{0}$the diffusivity,$\gamma_{h}$the proton gyromagnetic ratio,δthe diffusion gradient duration, and$g_{exp}$the diffusion gradient amplitude. Given that our experimental data were acquired on a 7T preclinical scanner with a gradient amplitude of 625.5 mT/m, an SNR of 21 dB, and an approximated diffusivity of 0.35$\mu m^{2}/ms$,$d_{min}∼1.21\mu$m. While we could conclude of a systematic overestimation of axon diameters smaller than 1.21μmdue to the diffusion acquisition constraints, we should highlight that the model is not tasked with predicting each feature in isolation. Instead, it leverages the correlations between g-ratio and axon diameter distributions. In addition, the chosen regions for both pretraining and transfer learning display predominantly homogeneously distributed fibers. This structured prediction approach increases the model’s capacity to derive meaningful insights from this particular dataset. Moreover, the integration of multi-contrast data within our model offers a key advantage, as it leverages complementary information to provide a more comprehensive view of tissue microstructure. Machine learning has demonstrated its capability to capture complex relationships arising from multimodal techniques (Krones et al., 2025), as well as to enhance the resolution of low-resolution data (de Leeuw den Bouter et al., 2022;Lau et al., 2023). This inspires confidence in the model capacity to extract meaningful information from particularly challenging dataset. Nonetheless, further data and testing would be required to generalize this model to regions with more complex axon distributions and/or smaller axons.

In addition, using synthetic data for model pretraining while fine-tuning on real-world data introduces inherent challenges. Both the feature extraction from EM data and MR simulation rely on simplifying assumptions (Drobnjak et al., 2010,^2011^;Ianuş et al., 2013,^2016^;Zaimi et al., 2018), which may impact the model’s ability to capture fine-grained information in complex tissue structures. Additionally, the axons used for synthetic image generation come from a single source (Vuong et al., 2017), which may introduce species- and region-specific biases in the training data. While we expect the network to learn generalizable features during pretraining that are refined in the transfer learning stage, the pretrained model could be influenced by characteristics unique to the dog spinal cord, exhibiting a bias toward large axonal bundles. Indeed, the extracted distribution of axonal diameters in the dog spinal cord dataset is centered on 2.07μmwhile the mouse experimental data have a distribution of axonal diameters centered on 0.31μm. This size discrepancy between the pretraining and the fine tuning could affect the network’s ability to generalize to regions with smaller or more densely packed axons, potentially leading to overestimation or underrepresentation of microstructural features in regions dominated by fine axons. However, this limitation could be addressed in the future by incorporating additional EM sources that more closely match the target distributions, reducing the risk of overfitting to features specific to the original synthetic dataset.

Moreover, for efficiency, we opted for relatively simple MRI simulators, which fail to account for several effects present in experimental data. Key limitations include mismatches in the thickness between EM images and MRI slices, the absence of distortions and T2 decay in the synthetic diffusion images, and assumptions made regarding biophysical constants for the magnetization transfer (MT) model. We did not consider contributions of cell bodies or other non-extra-axonal compartments which could result in overestimations of axonal volume fraction or axon diameter as the model wrongly attributes some diffusion weighting to axonal compartments.

Despite these limitations, we chose to implement this training method due to the limited availability of large paired MRI/histology datasets. The results show that, even with the limitations in the simulation software, the networks are able to learn some useful features that are then fine-tuned with the experimental dataset. The presented approach can be greatly refined by using more advanced substrate simulators (Fieremans & Lee, 2018;Ginsburger et al., 2019;Lee et al., 2023;Villarreal-Haro et al., 2023) or more advanced MRI simulators (Li et al., 2019;Rafael-Patino et al., 2020).

Further limitations include the limited amount of experimental data and the restricted spatial coverage as we only performed the experiment on the mouse brain corpus callosum. Our approach was tested on a dataset comprising only nine excised mouse brains, with the evaluation of generalizability conducted on the same mice at different developmental time points. Although this demonstrates the feasibility of using transfer learning to estimate microstructural features from MRI data, the limited sample size restricts the ability to fully assess the model’s generalizability across different biological contexts such as different species or brain regions. Additionally, because of the way the EM extraction is performed, the MRI voxels and the corresponding ground truth might show some misalignment.

Finally, the way we considered the inputs to the model here makes it protocol-dependent, which limits its generalizability. To address this limitation, we propose two strategies: first, resampling the input to conform to a standardized protocol (Neher et al., 2015;Poulin et al., 2017); second, enhancing the model architecture to incorporate protocol information directly into the inputs. One promising approach is to add an additional head to the network dedicated to embed protocol characteristics. This head could utilize an attention mechanism to prioritize relevant features based on the specific protocol, facilitating better generalization across various imaging protocols similar to previous work on combining multimodal medical data using transformers (Raminedi et al., 2024;K. Zhang et al., 2024). This strategy would allow the model to learn distinct representations while maintaining consistency in feature extraction.

Our proposed methodology serves as a stepping stone for researchers, enabling them to leverage sparse data to progressively develop a shared, data-centric model for brain microstructure prediction. This approach can be adapted for use with other species or structures, allowing researchers to tailor it to their unique requirements. Moreover, it resonates with the need for collaborative efforts in utilizing valuable data, as emphasized by the BigMac Dataset (Howard et al., 2023). By spearheading the creation of an ML framework to predict microstructure from scarce experimental data, we aim to encourage the development of a foundational model that enhances the handling of various protocols for improved brain modeling. This collaborative foundation could ultimately lead to greater accuracy and reliability in microstructural analyses, driving forward the field of brain research.

Future avenues for improvement include combining more realistic synthetic image generation for improved pretraining of the network as well as obtaining higher quality and more varied experimental data, for example by combining MRI with 3D histology (Chang et al., 2017;Goubran et al., 2019).

Incorporating auxiliary strategies, such as prior knowledge using biophysical maps and multi-task learning, serves as a pragmatic approach to enhance model performance in the prediction of brain microstructure. These techniques enable the model to capture complex relationships within multimodal data and address the inherent challenges posed by limited labeled examples. By leveraging complementary information, these strategies not only improve accuracy but also provide a more comprehensive view of the tissue microstructure. However, it is essential to recognize the need for developing models that can achieve similar or improved performance with fewer auxiliary methods, thus paving the way for more foundational models. This evolution will facilitate greater generalizability and applicability across different species and experimental conditions. Future work will focus on refining these methods and exploring alternative strategies—such as self-supervised or semi-supervised learning—to strengthen model robustness while minimizing reliance on auxiliary inputs.

## 5 Conclusions

We have presented a novel approach for microstructure prediction from raw multi-contrast MRI based on transfer learning. We showed the benefits of combining pretraining of a neural network in histology-based synthetic MRI data and a fully synthetic-substrate approach. Quantitative evaluations in a mouse brain experimental dataset showed Jensen-Shannon Divergences of 0.26 and 0.23 in axon diameter and g-ratio, respectively. These results suggest that this approach of pretraining with synthetic data can help alleviate the lack of big datasets containing both MRI and histological data. Further work will focus on enhancing the accuracy of the simulation steps and on looking at other microstructural features where this approach can be useful.

We consider the approach presented here is a first step toward having a hybrid, widely available model that includes previous biophysical model information with data-driven approaches that can be fine-tuned for specific applications. We consider that biophysical models should still be part of the model so as to provide a ground basis for the ML model, being it embedded in the structure or as additional information to the network, as is the case of this study. However, the goal of obtaining a foundation model for microstructural feature prediction will still require further studies and facing multiple challenges such as combining data from multiple sources, species, and anatomical regions, as well as making it compatible with different MRI inputs and not protocol-dependant. When properly transferred to human data, such a model could automate the extraction of microstructural characteristics, aiding in the diagnosis and monitoring of neurological disorders with less human input and faster computation compared to traditional biophysical model fitting.

## Data Availability

Code, data and trained models to replicate the results of the study are available athttps://github.com/emmweber/MRI_EM_brain_microstructure.

## Appendix

**Table:** Comparison of transfer learning performance metrics for models pretrained on baseline synthetic data derived from the dog spinal cord (baseline) and synthetic data with axon diameters scaled down by a factor of 0.15 (shrunk axons).

	Model	
Metric	Baseline	Shrunk axons
Genu (% error in mean)	4.32% ± 4.86%	3.62% ± 3.97%
Body (% error in mean)	2.79% ± 2.73%	3.43% ± 3.36%
Splenium (% error in mean)	3.02% ± 2.30%	4.35% ± 3.56 %
Genu effect size	1.76 ± 1.23	1.29 ± 1.08
Body effect size	3.02 ± 3.19	1.55 ± 1.68
Splenium effect size	-0.39 ± -0.29	0.06 ± 0.07
